# Comparison of Help-Seeking Consultations for Domestic Violence Before vs During the COVID-19 Pandemic in Japan

**DOI:** 10.1001/jamanetworkopen.2022.29421

**Published:** 2022-08-30

**Authors:** Xerxes T. Seposo

**Affiliations:** 1 Department of Hygiene, Faculty of Medicine, Graduate School of Medicine, Hokkaido University, Sapporo, Japan; 2 School of Tropical Medicine and Global Health, Nagasaki University, Nagasaki, Japan

## Abstract

This cross-sectional study compares rates of help-seeking consultations for incident domestic violence before vs during the COVID-19 pandemic in Japan.

## Introduction

Since 2011, rates of professional help-seeking consultations (ie, inquiries) for domestic violence (DV) have been increasing steadily in Japan, with a considerable upward trend during the COVID-19 pandemic.^1^ Pandemic-related restrictions have increased time spent in domestic settings and contributed to income instability for both perpetrators and survivors of DV.^2^ These living conditions affect households with potentially abusive individuals, with survivors of DV bearing most of the physical and mental health burden.^3^ We compared rates of incident DV inquiries during the COVID-19 pandemic with years before onset in Japan.

## Methods

Nagasaki University deemed this cross-sectional study exempt from ethics review, and informed consent was waived because publicly available aggregated and anonymized data were used. The study followed the STROBE reporting guideline.

In Japan, DV is defined as violence perpetrated by a spouse, sexual partner, or another person with whom one is or has been in an intimate relationship. Data for annual DV inquiries (2011-2020) for the 47 Japanese prefectures were obtained from Gender and Equality Bureau Cabinet Office of Japan online sources.^4^ Incident DV inquiry rates were calculated for individuals aged 15 to 64 years, including married and cohabiting couples, and are reported per reporting facility per 1000 population. The eMethods in the Supplement details the nature of DV inquiries, calculation of incident DV rates, and means of inquiry.

A paired 2-sample, Wilcoxon signed-rank test was used to determine differences in annualized prefecture-specific incident DV inquiry rates before (2011-2019) vs during (2020) the pandemic. Subgroup analyses were conducted for 2 help-seeking categories: sex and means of inquiry (facility based, call center based, and other). Significance was set at *P* < .05. Analyses were performed using R version 4.2.0 (R Foundation for Statistical Computing).

## Results

From 2011 to 2020, there were 1 061 410 DV inquiries in Japan. Of these, 1 041 558 (98.1%) were from female individuals and 19 852 (1.9%) were from male individuals. The majority of inquiries were made through call centers (700 683 [66.0%]), followed by in-facility visits (318 946 [30.0%]) and other means (41 781 [4.0%]). Annual incident DV inquiry rates were 32.0 and 38.0 per reporting facility per 1000 population before vs during the pandemic, respectively. In 2020, incident DV inquiry rates were higher for female individuals (125 916 [97.2%]) compared with male individuals (3575 [2.8%]; Table). Among means of inquiry, only call center–based inquiries increased significantly during the pandemic (68 279 [65.9%] annualized in 2011-2019 vs 86 168 [66.5%] in 2020; *P* < .001).

**Table.** Subgroup Analysis of Domestic Violence Summary Characteristics Before vs During the COVID-19 Pandemic in Japan

Characteristic	No. of inquiries, %			*P* value^c^
	Before pandemic total (n = 931 919)^a^	Before pandemic annualized (n = 103 547)^b^	During pandemic total (n = 129 491)	
Sex				
Male	16 277 (1.7)	1809 (1.7)	3575 (2.8)	<.001
Female	915 642 (98.3)	101 738 (98.3)	125 916 (97.2)	.03
Means of inquiry				
In-facility visit	281 035 (30.2)	31 226 (30.2)	37 911 (29.3)	.43
Call center	614 515 (65.9)	68 280 (65.9)	86 168 (66.5)	<.001
Other	36 369 (3.9)	4041 (3.9)	5412 (4.2)	.14

^a^ The prepandemic period includes data from 2011 to 2019, whereas the COVID-19 pandemic period includes 2020 only.

^b^ Data from 2011 to 2019 were annualized.

^c^ *P* values were calculated using the paired 2-sample, Wilcoxon signed-rank test with prefecture-specific inquiry rates before vs during the pandemic. *P* = .02 for analyses of help-seeking categories of sex and means of inquiry.

Sex and means of inquiry–specific incident DV inquiries have risen since 2011 in Japan, with a sharp increase in 2020 (Figure). Further examination revealed a significant increase (*P* = .02) between study periods for both the sex and means of inquiry help-seeking categories, with a higher nationwide rate during the pandemic (129 491 in 2020) vs before (103 547 annualized in 2011-2019; Table).

**Figure.**
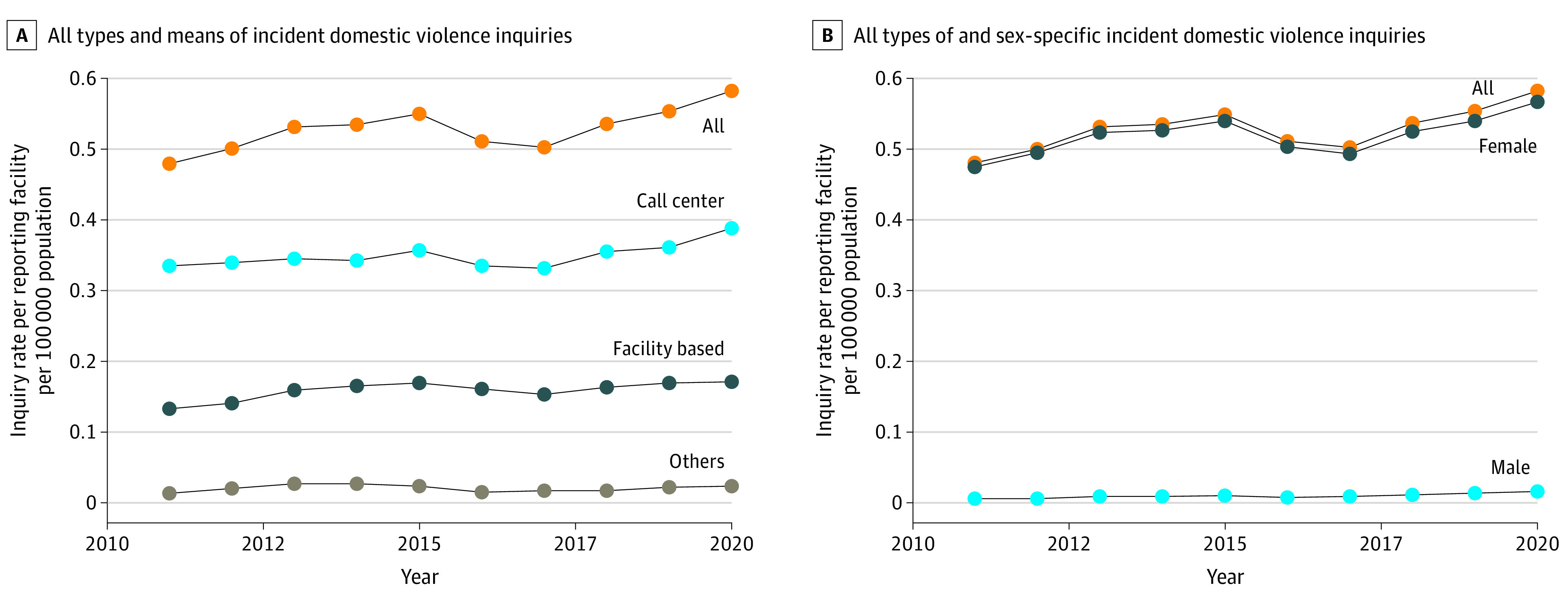
Domestic Violence Summary Characteristics Before (2011-2019) vs During (2020) the COVID-19 Pandemic in Japan A, All types and means of incident domestic violence professional help-seeking inquiries. B, All types of and sex-specific incident domestic violence inquiries.

## Discussion

This cross-sectional study found that incident DV inquiries in Japan increased between 2011 and 2020. This increase may be associated with a range of factors, including economic instability, increased exposure to exploitative relationships, and reduced support options.^2,5^ Previous studies note that COVID-19 restrictions have cultivated an environment in which perpetrators of DV exhibit tendencies of dominant control.^1,5^ Bhandari et al^3^ recently reported that physical abuse was associated with the state-of-emergency declaration in Japan. They further observed that the incidence of physical abuse was notably higher among female individuals, similar to observations in this study. In the US, DV-related arrests and calls pertaining to family violence have increased during the pandemic compared with similar periods before onset.^6^

This study has several limitations. First, data for individuals aged 20 years or older and the proportion of cohabiting couples are needed for increased precision. Second, data with coverage beyond 2020 may provide additional insight regarding the effects of the pandemic on DV inquiries. Additionally, caution should be exercised in generalizing the results for Japan to other countries.

## Notes

Supplement.
eMethods
