# Supplementary material for: Comparison of Help-Seeking Consultations for Domestic Violence Before vs During the COVID-19 Pandemic in Japan
Source: JAMA Netw Open. 2022 Aug 30;5(8):e2229421. doi: 10.1001/jamanetworkopen.2022.29421 (PMC9428737; doi:10.1001/jamanetworkopen.2022.29421)
Supplement: Supplement. — eMethods [file jamanetwopen-e2229421-s001.pdf]

Supplementary Online Content

Seposo XT. Comparison of help-seeking consultations for domestic violence before vs during the COVID-19 pandemic in Japan. *JAMA Netw Open*. 2022;5(8):e2229421. doi:10.1001/jamanetworkopen.2022.29421

eMethods

This supplementary material has been provided by the authors to give readers additional information about their work.

© 2022 Seposo XT. *JAMA Network Open*.

eMethods

Nature of Domestic Violence (DV) consultations

In Japan, DV consultations are monitored, maintained, and reported by the Gender and Equality Bureau Cabinet Office of the Japanese Government via the Spousal Violence Counseling and Support Centers, which began operation in each prefecture on April 1, 2002 (espoused under the Law for the Prevention of Spousal Violence and the Protection of Victims was enacted in April 2001 and came into effect on October 13, 2001 (or April 1, 2002, for some cases).

These specific consultations are dedicated to domestic violence and other non-DV consultations usually course through the police and other entities separately handling the cases. This is a unique system solely dedicated to DV concerns in Japan. Consultations vary from enquiries to potentially life-threatening intimidation. The system does not include medical consultations, which are separately monitored and maintained by relevant hospitals or clinics. These enquiries refer to the number of times when an enquiry was recorded in the reporting facility and not the number of unique person who enquired. Furthermore, these (enquiries) may or may not lead to actual formal complaints of DV cases.

Incident DV enquiry calculation

Prefecture-specific, year-specific DV enquiries were transformed into "prefecture-specific (*i*), year-specific (*j*), incident DV enquiries per reporting facility, per 1,000 population by using Supplementary Equation 1 (below).

$$Incident\ DV\ enquiries\ \ per\ sentinel, per\ 1{,}000\ population_{i,j} = \left(\frac{\frac{No.\ of\ annual\ DV\ enquiries_{i,j}}{No. of\ reporting facility_{i,j}}}{annual\ population_{15-64\ years\ old_{i,j}}}\right) \times 1{,}000\ population \quad [SEq1]$$

15-64 old population was used as a denominator to capture both married and cohabiting couples, and potentially exclude the adolescent and children population (less than 20 years old). Cases

© 2022 Seposo XT. *JAMA Network Open*.

involving abuse of adolescents and children are classified as child abuse and are separately monitored.

Means of Enquiry

In brief, in-facility enquiries are visitation to the Spousal Violence Counseling and Support Centers, which are located within the respective locations (ward, city, and prefecture levels). A complete list of these locations can be accessed through the following URL: https://www.gender.go.jp/policy/no_violence/e-vaw/soudankikan/pdf/center.pdf. Whereas call-center-based services are accessed via dedicated hotlines (0570-0-55210 or #-8-0-0-8) using either a mobile phone or a landline phone. Other means of enquiry are through electronic mail.

© 2022 Seposo XT. *JAMA Network Open*.
